# Henrin A: A New Anti-HIV *Ent*-Kaurane Diterpene from *Pteris henryi*

**DOI:** 10.3390/ijms161126071

**Published:** 2015-11-24

**Authors:** Wan-Fei Li, Juan Wang, Jing-Jie Zhang, Xun Song, Chuen-Fai Ku, Juan Zou, Ji-Xin Li, Li-Jun Rong, Lu-Tai Pan, Hong-Jie Zhang

**Affiliations:** 1 Guiyang College of Traditional Chinese Medicine, Guiyang 550002, China; wanfeier@live.cn (W.-F.L.); zjj523@126.com (J.-J.Z.); zoujuan-729@163.com (J.Z.); lijixinmylove@yeah.net (J.-X.L.); 2 School of Chinese Medicine, Hong Kong Baptist University, 7 Baptist University Road, Kowloon Tong, Kowloon, Hong Kong, China; juan_wang2012@163.com (J.W.); kobe24song@126.com (X.S.); faiku2010@hkbu.edu.hk (C.-F.K.); 3 School of Public Health, Jilin University, Changchun 130021, China; 4 Department of Microbiology and Immunology, College of Medicine, University of Illinois at Chicago, 833 South Wood Street, Chicago, IL 60612, USA; lijun@uic.edu

**Keywords:** *Pteris henryi*, *ent*-kaurane diterpene, henrin A, bioactivity evaluation, anti-HIV activity

## Abstract

Henrin A (**1**), a new *ent*-kaurane diterpene, was isolated from the leaves of *Pteris henryi*. The chemical structure was elucidated by analysis of the spectroscopic data including one-dimensional (1D) and two-dimensional (2D) NMR spectra, and was further confirmed by X-ray crystallographic analysis. The compound was evaluated for its biological activities against a panel of cancer cell lines, dental bacterial biofilm formation, and HIV. It displayed anti-HIV potential with an IC_50_ value of 9.1 µM (SI = 12.2).

## 1. Introduction

*Ent*-kaurane compounds are members of a class of diterpenes with a four-membered ring system, which are richly found in the *Isodon* genus (Lamiaceae) [1,2,3]. They have been known for having a variety of biological activities, including anticancer and antibacterial activities [4,5,6]. Tetracyclic *ent*-kauranes have also been found in the fern plants of the genus *Pteris* (Pteridaceae family), and some of them have demonstrated biological activities [7,8,9,10,11,12,13]. Phytochemical and biological investigation of the plants in the genus *Pteris* may produce potentially novel bioactive diterpenes [14,15].

**Figure 1:**
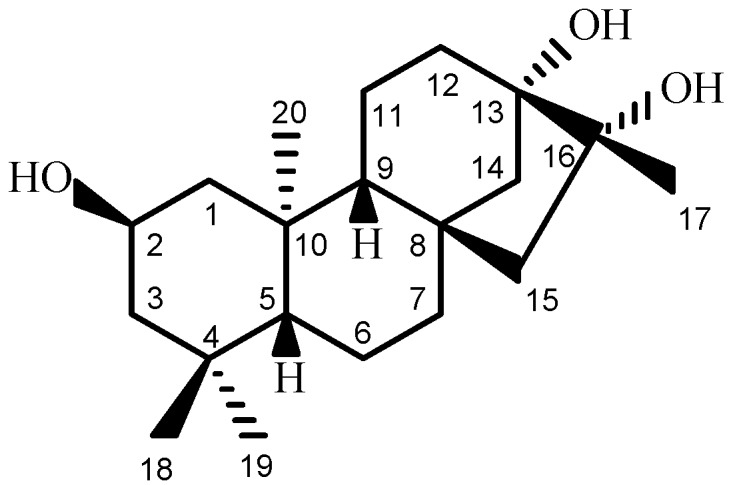
Structure of compound henrin A (**1**).

The genus *Pteris* comprises more than 300 species, 66 of which are distributed in China [16]. Few phytochemical studies have been reported for the chemical constituents of the plants in this genus [17]. Our present study focused on the plant species *P. henryi* Chirst, a perennial herb that has been used as an herbal medicine for the treatment of burns and scalds, lyssodexis, traumatic hemorrhages, leucorrhea, and difficulty and pain in micturition [18]. The plant is mainly distributed in the Guizhou and Yunnan provinces, People’s Republic of China [19]. In this study, we report the isolation, structural determination, and biological activity evaluation of henrin A (**1**), a new *ent*-kaurane diterpene (Figure 1). 

## 2. Results and Discussion

### 2.1. Compound Identification

The dried leaves of *P. henryi* were extracted with MeOH to afford a MeOH extract, which was separated through silica gel chromatography to yield henrin A (**1**).

Henrin A (**1**) was obtained as colorless crystals with UV (MeOH) λ_max_ (log ε) at 204 (1.60) nm (Figure S1). In the IR spectrum, **1** showed absorption of hydroxyl groups (3386, 1099, and 1049 cm^−1^) (Figure S2). Its molecular formula was determined to be C_20_H_34_O_3_ by means of analyzing its NMR spectroscopic data (Table 1), and further verified by the HR-EIMS data with *m/z* 345.2405 [M + Na]^+^ (calcd 345.2400) (Figure S3). The molecule of **1** has four double-bond equivalences. However, no carbonyl absorption was observed in the IR spectrum. As evidenced from the ^1^H and ^13^C NMR spectral data (Table 1) (Figures S4–S6) as well as the HMQC correlation data (Figure S7), the 20 carbons of compound **1** were characterized as four methyl groups (δ_H_ 0.87, 0.93,1.09, and 1.18 (each 3H, s); δ_C_ 19.4 (q), 21.3 (q), 22.8 (q), and 34.2 (q)), an oxy-methine group (δ_H_ 3.87 (1H, brtt, *J* = 11.5, 4.3 Hz); δ_C_ 65.4 (d)), two oxy-tertiary carbons (δ_C_ 77.4 (s) and 81.1 (s)), eight methylene carbons, two non-oxygenated methine carbons (δ_H_ 0.82 (1H, brd, *J* = 11.5 Hz), and 0.96 (1H, brd, *J* = 7.2 Hz); δ_C_ 57.0 (d) and 57.1 (d)), and three quaternary carbons. No signals were observed in the range of δ_H_ 5–7 ppm of the ^1^H NMR spectrum and in the range of δ_C_ 90–160 ppm of the ^13^C NMR spectrum, indicating that there is no carbon-carbon double-bond in **1**. The lack of olefinic signals in the molecule and the calculation of four double-bond equivalences determined that **1** has a tetracyclic-ring system. Compound **1** was thus suggested to have a saturated tetracyclic diterpene having an *ent*-kaurane skeleton.

**Figure 2:**
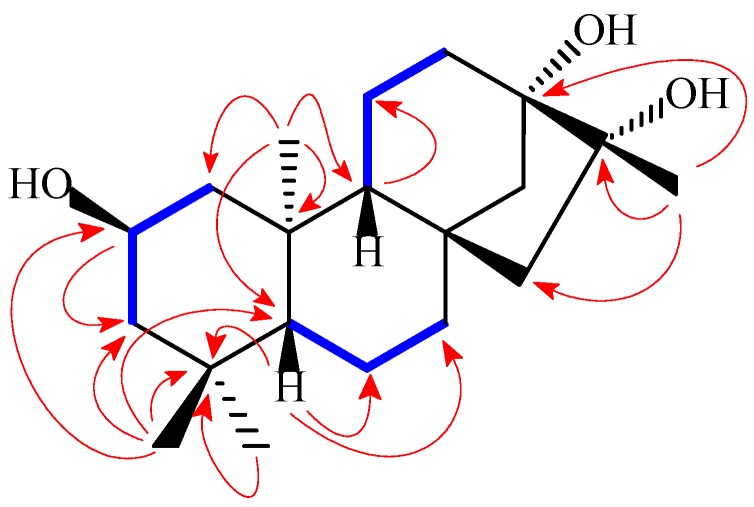
Key COSY (marked as blue bold bonds (H
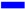
H)) and HMBC (the red arrows (H
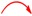
C)) correlations for compound **1**.

Through analysis of the long-range correlation data observed in the HMBC (heteronuclear multiple-bond correlation spectroscopy) spectrum (Figure S8), together with the HMQC (heteronuclear multiple-quantum correlation spectroscopy) (Figure S7) and ^1^H–^1^H COSY (correlated spectroscopy) (Figure S9) data (Figure 2), the three oxy-carbon groups (one oxy-methine and two oxy-tertiary carbons) in **1** could be assigned accordingly. Starting from the singlet signals of the methyl protons at C-18 and C-19 (δ_H_ 0.93 (H_3_-18), 0.87 (H_3_-19)), the ^13^C NMR at δ_C_ 51.9 (t) was assigned to C-3 due to the presence of its HMBC correlations to the two methyl protons, which in turn suggested the oxy-methine group at C-2 due to the presence of the ^1^H–^1^H COSY correlations between H-2 (δ_H_ 3.87) and H_2_-3 (δ_H_ 1.07 and 1.72). The presence of the HMBC correlations of the singlet signals of the methyl protons at C-17 (δ_H_ 1.18) to both oxy-tertiary carbons at δ_C_ 77.4 and 81.1 suggested that both C-13 and C-16 were subsitituted with a hydroxy group, respectively.

**Figure 3:**
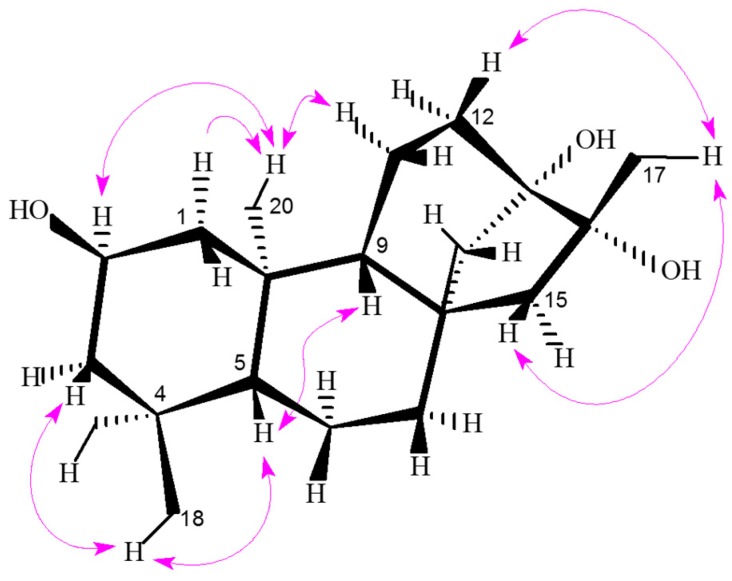
Key ROESY correlations (indicated as magenta arrows (H
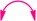
H)) for compound **1**.

In the ROESY (rotating frame nuclear Overhauser effect spectroscopy) spectrum (Figure S10), the presence of the ROE (rotating frame Overhauser effect) correlations (Figure 3) of H-2 (δ_H_ 3.87) with H_2_-20 suggested the hydroxy group of C-2 to be β-oriented. In addition, the presence of the ROE correlations of H-5 (δ_H_ 0.82) to H_3_-18 and the lack of ROE cross-peaks of H-5 to H-2 and H_3_-19 suggested H-5 to be β-oriented. Furthermore, the β-orientation of H-9 (δ_H_ 0.96) was deduced on the basis of the observation of the ROE correlation of H-9 with H-5 and the lack of ROE correlation between H-9 and H_3_-20.

**Table 1:** ^1^H and ^13^C NMR data of compound **1** *^a^*.

No.	δ_H_ (mult, *J* in Hz) *^b^*	δ_C_ (mult) *^c^*	No.	δ_H_ (mult, *J* in Hz) *^b^*	δ_C_ (mult) *^c^*
1α	2.15 (brddd, 12.0, 3.8, 1.9)	50.3 t	11α	1.65 (overlap)	20.7 t
1β	0.67 (brt, 11.7)	-	11β	1.82 (overlap)	-
2α	3.87 (brtt, 11.5, 4.3)	65.4 d	12α	1.68 (overlap)	34.6 t
3α	1.72 (brddd, 12.5, 4.3, 1.9)	51.9 t	12β	1.64 (overlap)	-
3β	1.07 (brt, 12.0)	-	13	-	77.4 s
4	-	35.7 s	14α	1.66 (overlap)	44.0 t
5β	0.82 (brd, 11.5)	57.0 d	14β	1.83 (d, 11.9)	-
6α	1.34 (brqd, 12.1, 1.9)	21.2 t	15α	1.50 (dd, 14.5, 1.3)	56.7 t
6β	1.59 (brd, 12.3)	-	15β	1.63 (d, 14.7)	-
7α	1.60 (overlap)	43.2 t	16	-	81.1 s
7β	1.44 (brtd, 11.9, 3.4)	-	17	1.18 (s)	21.3 q
8	-	42.2 s	18	0.93 (s)	34.2 q
9β	0.96 (brd, 7.2)	57.1 d	19	0.87 (s)	22.8 q
10	-	41.9 s	20	1.09 (s)	19.4 q

*^a^* Data were recorded in CD_3_OD; δ values are given in ppm with reference to the signal of CD_3_OD (δ 3.31 ppm) for ^1^H and to the center peak of the signal of CD_3_OD (δ 49.0 ppm) for ^13^C; *^b^* Multiplicities in parentheses represent: s (singlet), brs (broad singlet), dd (doublet of doublet), brdd (broad doublet of doublet), brtd (broad triplet of doublet), brqd (broad quartet of doublet), ddd (doublet of doublet of doublet), brddd (broad doublet of doublet of doublet), t (triplet), brt (broad triplet), and brtt (broad triplet of triplet); *^c^* Multiplicities represent: s (quaternary carbon), d (CH), t (CH_2_), and q (CH_3_).

**Figure 4:**
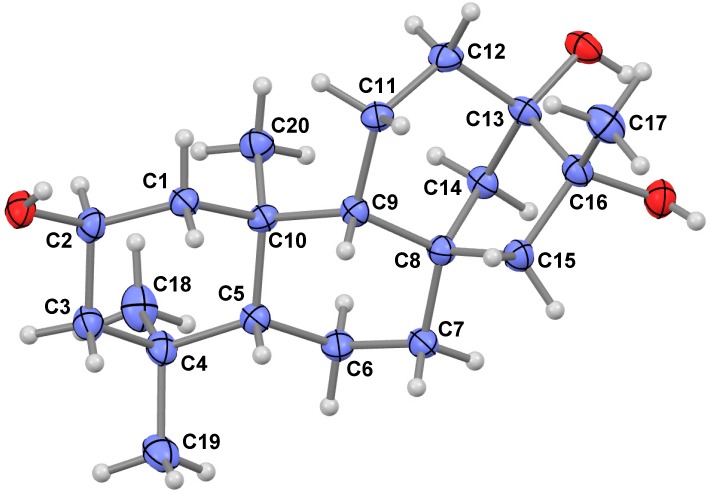
ORTEP (oak ridge thermal ellipsoid plot program) drawing of compound **1** (Blue ball: carbon; grey ball: hydrogen; red ball: oxygen).

To further confirm the chemical structure, compound **1** was crystallized in MeOH to afford a colorless crystal with the monoclinic space group of P2_1_2_1_2_1_, which was analyzed by X-ray crystallography. Through structural refinement [20,21], the chemical structure of **1** was confirmed as shown in Figure 4. Like the *Isodon* plants, the diterpenes produced by the ferns in the genus *Pteris* are all *ent*-kaurane diterpenes [7,8,9,10,11,12,13]. Compound **1** is also an *ent*-kaurane diterpene due to the observation of the negative optical rotation (${\left[\text{α}\right]}_{D}^{11}$ − 5.66°) as well as the consideration of the similar biogenetic pathways used by the *Pteris* plants for producing the same class of congeners [22,23,24]. Accordingly, **1** was determined to be *ent*-2β, l3α, 16α-trihydroxy-kaurane, and given the trivial name of henrin A.

### 2.2. Biological Activity

Henrin A (**1**) was evaluated for its cytotoxic activity against a panel of human cancer cell lines comprising KB (cervical), HCT116 (colon), A549 (lung), and MCF-7 (breast) cell lines. No inhibitory activity against these cell lines was observed for **1** at a concetration of 20 µg/mL. Due to the low cytotoxicity of the compound, **1** was further evaluated for its antimicrobial potential. 

The dental biofilm formation inhibitory as well as the antifungal activity assays (Table 2) determined that **1** had no antimicrobial activity against the two dental pathogens *Streptococcus mutans* and *S. sobrinus* at a concetration of 20 µg/mL. Compound **1** was also evaluated for its antifungal activity against the athelete’s foot fungus *Trichophyton rubrum*. No antifungal inhibitory activity was observed for this compound at a concentration of 20 µg/mL (Table 2).

**Table 2:** Antimicrobial activity of henrin A (**1**) against biofilm of two dental bacteria and the athelete’s foot fungus.

Compounds	Growth Inhibition Rate (%)		
	Bacterial Biofilm Formation		Fungus
	*S. mutans*	*S. sobrinus*	*T. rubrum*
Henrin A (1) *^a^*	15.55 ± 5.66	11.20 ± 2.23	9.73 ± 8.75
Penicillin G *^b^*	80.44 ± 3.14	76.82 ± 3.93	-
Chlorhexidine *^c^*	77.26 ± 6.30	71.49 ± 5.21	83.12 ± 0.47
Miconazole *^a^*	-	-	80.69 ± 0.10

*^a^* Compound assay concentration at 20 µg/mL; *^b^* Compound assay concentration at 10 µg/mL; *^c^* Compound assay concentration at 12 µg/mL.

**Figure 5:**
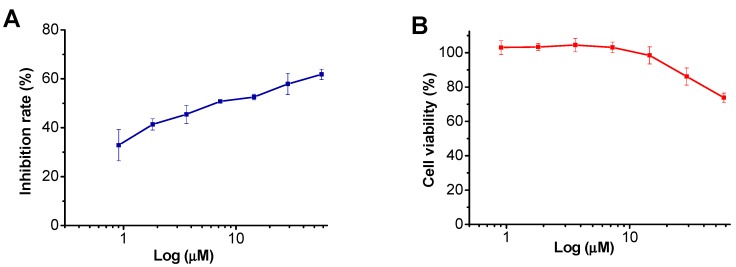
Inhibition of HIV/VSV-G by compound **1**. (**A**) The inhibitory effect of **1** on HIV/VSV-G infectivity was investigated, and it displayed dose-dependent inhibitory activities; (**B**) The cytotoxicity of **1** was tested on A549 target cells. Three independent experiments were performed to determine the effect of the compound.

Henrin A (**1**) was then evaluated for its anti-HIV activity using our previously established “One-Stone-Two-Birds” assay evaluation system [25]. This protocol allows us to identify potential inhibitors for HIV replication (post-entry steps). This protocol is an easy, safe, and efficient HIV vector-based assay system to evaluate and identify potential inhibitors against HIV replication (Figure 5 and Table 3). Henrin A (**1**) was found to exhibit anti-HIV activity with an IC_50_ value of 9.1 µM with selective index of 12.2.

**Table 3:** Anti-HIV activity of compound **1**.

Compound	IC_50_ (µM)	CC_50_ (µM)	SI
Henrin A	9.1	110.5	12.2
AZT (zidovudine)	0.03	>100	>3333

Many kaurane compounds have been found to possess cytotoxic activity due to the presence of an α, β-unsaturated ketone group in their structures [26]. However, for those without this structural feature, low or no cytotoxicity activity is expected [27,28,29,30], and these compounds thus may be further explored for their antimicrobial potential. Henrin A (**1**) is an *ent*-kaurane diterpene belonging to this category, and our assay determined that this compound was not toxic against a panel of human cell lines but indeed displayed anti-HIV activity. More than 1000 different types of *ent*-kaurane compounds have been discovered from plants [30]. These diterpenes can be a valuable source for the discovery of antiviral agents by testing the natural or modified compounds without an α, β-unsaturated ketone group.

## 3. Experimental Section

### 3.1. General Experimental Procedures

Optical rotation was measured with a Rudolph digital polarimeter. IR spectrum was recorded on a VECTOR22 spectrophotometer (Bruker, Rheinstetten, Germany) with KBr pellets. 1D (one dimentiona) and 2D (two dimentional) NMR spectra were recorded on a JEOL 500MHz spectrometer (JEOL Ltd., Tokyo, Japan). Unless otherwise specified, chemical shifts (δ) were expressed in ppm with reference to the solvent signals. High-resolution mass spectrum (HR-EIMS) was performed on a VG Autospec-3000 spectrometer (VG, Manchester, UK) under 70 eV. Column chromatography was performed with silica gel (200–300 mesh; Qingdao Marine Chemical, Inc., Qingdao, China). Fractions were monitored by TLC and spots were visualized by heating silica gel plates sprayed with 10% H_2_SO_4_ in EtOH. All solvents including petroleum ether (60–90 °C) were distilled prior to use. 

### 3.2. Plant Material

The plant materials of the leaves of *P. henryi* Chirst were collected in Anshun, Guizhou Province, China, in September 2010. The voucher specimen was identified by Professor Junhua Zhao of the Guiyang College of Traditional Chinese Medicine, and deposited at Guiyang College of Traditional Chinese Medicine, with the number of the voucher specimen as No. 20101003. 

### 3.3. Extraction and Isolation

The air-dried powder of the leaves of *P. henryi* Chirst (5 kg) was percolated with 95% MeOH at room temperature (3 × 5 L), and the MeOH crude extract (266 g) was subjected to silica gel chromatography separation (10 cm × 100 cm), eluting with a gradient solvent system of CHCl_3_/MeOH (from 10/1 to 0/1, *v*/*v*) to afford six fractions (A–F). Fraction C was separated over an additional chromatographic column of silica gel (30 mm × 300 mm), eluting with a gradient solvent system of CHCl_3_/MeOH (from 5/1 to 1/1, *v*/*v*) to afford fraction G, which was further separated by a Sephadex LH-20 column (300 mm × 1000 mm), eluding with the solvent CHCl_3_/MeOH (1/1, *v*/*v*) to afford herin A (30 mg).

Henrin A (**1**): Colorless crystals (MeOH); mp 246–248 °C; ${\left[\text{α}\right]}_{D}^{11}$ − 5.66° (*c* 2.12, MeOH); UV (MeOH) λ_max_ (log ε) 204 (1.60) nm; IR (KBr) ν_max_ 3386, 2940, 2865, 1447, 1468, 1334, 1146, 1099, 1049, 601 cm^−1^; ^1^H and ^13^C NMR, see Table 1; HR-EIMS ([M]^+^ *m/z* 345.2405 [M + Na]^+^ (calcd. 345.2400 for C_20_H_34_O_3_Na)).

### 3.4. X-ray Data of Henrin A (**1**)

CCDC 1039988 contains the supplementary crystallographic data for this paper. These data can be obtained free of charge from The Cambridge Crystallographic Data Centre (Available online: www.ccdc.cam.ac.uk/data_request/cif). Crystal data of **1** (from MeOH): space group P2_1_2_1_2_1_, C_20_H_34_O_3_, M = 322.25, *a* = 6.462(2) Å, *b* = 12.320(4) Å, *c* = 22.311(7) Å, α = β = γ = 90.00°, *V* = 1776.3(9) Å^3^, *T* = 293(2) K, Z = 4. μ (Mo *K*α) = 0.71073 mm^−1^. A crystal of dimensions of 0.26 mm × 0.25 mm × 0.24 mm was used for measurements on an APEX DUO diffractometer (Bruker, Rheinstetten, Germany) with a graphite monochromator (ω-κ scans, 2θ_max_ = 50.00°), Mo *K*α radiation. The total number of independent reflections measured was 3113, of which 2250 were observed (|F|^2^ ≥ 2σ|F|^2^). The crystal structure was solved and refined by the direct method SHELXS-97 [31], expanded using difference Fourier techniques and full-matrix least-squares calculations. Final indices: R1 = 0.0601, wR2 = 0.1458 (w = 1/σ|F|^2^), s = 1.062. Flack parameter = −0.3(2).

### 3.5. Biological Activity of Henrin A (**1**)

#### 3.5.1. Cytotoxicity Assay

Cytotoxicity assays involving oral epidermoid (KB: a Hela derivative previously referred as oral epidermis), colon (HCT116), breast (MCF-7), and lung (A549) carcinoma cell lines (ATCC, Manassas, VA, USA) were performed using sulforhodamine B based on the slightly modified protocols used for individual cell lines [32]. KB and A549 cells were maintained in Dulbecco's modified Eagle’s medium (DMEM) (Life Technologies, Carlsbad, CA, USA). HCT116 cells were maintained in McCoy’s 5A medium (Life Technologies). MCF-7 cells were maintained in DMEM medium containing 10 mg/L of insulin. Briefly, medium was supplemented with 10% fetal bovine serum (FBS) (Life Technologies). Serial dilutions of test samples were prepared using 10% aqueous DMSO as solvent. The cell suspension was added into 96-well microliter plates in 190 µL at plating densities of 5000 cells/well. One plate was fixed *in situ* with TCA to represent a no growth control at the time of drug addition (day 0). Then 10 μL 10% aqueous DMSO was used as control group. After 72 h incubation, the cells were fixed to plastic substratum by the addition of 50 μL cold 50% aqueous trichloroacetic acid and washed with water after incubation at 4 °C for 30 min. After staining cells with 100 μL of 0.4% sulforhodamine B in 1% aqueous AcOH for 30 min, unbound dye was removed by washing four times with 1% aqueous AcOH. Allowed the plates to dry at room temperature, then the bound dye was solubilized with 200 μL 10 mM unbuffered Tris base, pH 10. Shaken for 5 min or until the dye was completely solubilized and the optical density was measured at 515 nm using an ELISA plate reader (Bio-Rad, Hercules, CA, USA). The average data were expressed as a percentage, relative to the control. Percentage growth inhibition was calculated as: (OD (cells + samples) − OD (day 0 only cells))/(OD (cells + 10% DMSO) − OD (day 0 only cells)) = % survival, Cytotoxicity = 1 − % survival.

#### 3.5.2. Anti-Biofilm Activity Test

The anti-dental bacterial activity was evaluated against *Streptococcus mutans* (ATCC35668) and *S. sobrinus* (ATCC33478) (both strains were obtained from Rory Munro Watt’s laborary of Faculty of Dentistry, Hong Kong University, Hong Kong), and the antifungal activity was evaluated against *Trichophyton rubrum* (ATCC MYA-4438) (obtained from Institute of Dermatology, Chinese Academy of Medical Science, Nanjing, China). Biofilm formation was quantified according to a method previously described [33], with minor modifications. Briefly, the bacteria were suspended in BHI broth until turbidity was equal to a 0.5 McFarland Standard [34], and then bacterial suspension was diluted 1:100 into fresh BHI broth in microtiter wells (SPL Lifesciences Co., Gyeonggi-do, Korea) supplemented with the compound at a final concentration of 20 μg/mL or with penicillin G, chlorhexidine, and DMSO as the two positive and one negative controls, respectively. After 24 h of incubation at 37 °C without agitation, the content of each well was removed, and each well was washed three times with 250 μL of sterile physiological saline. The plates were vigorously shaken in order to remove all non-adherent bacteria. The remaining attached bacteria were fixed with 200 μL of methanol per well, and after 15 min plates were emptied and left to dry. Then, 50 μL of the crystal violet solution (1%, *wt*/*vol*) was added to each sample well, and the mixture was incubated at room temperature for 15 min. Wells were washed four times with distilled water and were filled with 200 μL of 95% ethanol to solubilize crystal violet in the solvent. The eluent (150 μL) was transferred to a new microtiter well, and the absorbance was determined with a multimode microplate reader (Bio-Rad) at 570 nm.

#### 3.5.3. Antifungal Microtiter Assay

A 96-flat-bottom-well microtiter plate (SPL Lifesciences Co., Gyeonggi-do, Korea) was used in experiments to evaluate the effectiveness of the compound in inhibiting *T. rubrum*. The antifungal activity tests were performed using the broth micro dilution method as described in M38-A2, with modifications [35,36]. The medium used was Roswell Park Memorial Institute medium (RPMI) 1640 (Life Technologies Inc., Grand Island, NE, USA) with l-glutamine buffered to pH 7.0 with 0.165 M morpholinepropanesulfonic acid (MOPS), supplemented with 2% glucose. The cell suspension was prepared in growth medium. Each test well received 190 µL of conidia at 2.0 × 10^4^/mL, and 10 µL of the compound solution at 4 mg/mL, where the final concentrations in the well were 20 μg/mL. Positive (10 μL of miconazole with 190 μL of inoculum) and negative (200 μL of medium) controls were included in all experiments. The plates were incubated at 30 °C for 72 h. Then, 20 μL 5 mg/mL 3-(4,5-dimethylthiazol-2-yl)-2, 5-diphenyltetrazolium bromide (MTT) was added into the 96-well plate, then cultured 10 more hours. The pate was centrifuged at 2500 rpm for 10 min. Remove the supernatant and add 150 μL DMSO to dissolve the formazan by shaking for 30 min. The 96-well plate was centrifuged and the supernatant was transferred to a new 96-well plate, and OD reading was measured at 510 nm by using the microplate reader (Bio-Rad).

#### 3.5.4. Inhibitory HIV Activity Assay

HIV/VSV-G were produced by co-transfecting 3 g of VSV-G envelope expression plasmid with 21 g of a replication-defective HIV vector (pNL4-3.Luc.R∙E) [37,38] into human embryonic kidney 293T cells (90% confluent) in 10 cm plates with PEI (polyethylenimine) (Invitrogen, Carlsbad, CA, USA), as previously described [26]. Eight hours post-transfection, all media was replaced with fresh, complete DMEM. Forty-eight hours post-transfection, the supernatants were collected and filtered through a 0.45-µm-pore-size filter (Millipore, Billerica, MA, USA) and the pseudovirions were directly used for infection.

Target A549 cells were seeded at 10^4^ cells per well (96-well plate) in complete DMEM. Ten microliter compound for serial concentrations (20, 10, 5, 2.5, 1.25, 0.625, and 0.3125 µg/mL) and 190 µL of the pseudovirus were incubated with target cells. Forty-eight hours post-infection, cells were lysed and prepared for luciferase assay (Promega, Madison, WI, USA).

## 4. Conclusions

A new *ent*-kaurane diterpene (henrin A, **1**) was isolated from the leaves of *P. henryi*. The chemical structure was elucidated by analysis of the spectroscopic data and was further confirmed by the X-ray crystallographic analysis. The compound was evaluated for its biological activities against a panel of cancer cell lines, dental bacterial biofilm formation, and HIV. Our assay results showed that henrin A (**1**) had low cytotoxicity due to its lack of an α,β-unsaturated ketone group in the structure, but the compound has been determined as a potential anti-HIV agent in our antiviral assay study.

## Supplementary Materials

Supplementary materials can be found at http://www.mdpi.com/1422-0067/16/11/26071/s1.
