# Supplementary material for: Henrin A: A New Anti-HIV Ent-Kaurane Diterpene from Pteris henryi
Source: Int J Mol Sci. 2015 Nov 24;16(11):27978–87. doi: 10.3390/ijms161126071 (PMC4661929; doi:10.3390/ijms161126071)
Supplement: Supplementary file 1 [file ijms-16-26071-s001.pdf]

Supplementary Materials: Henrin A: A New Anti-HIV *Ent*-Kaurane Diterpene from *Pteris henryi*

Wan-Fei Li, Juan Wang, Jing-Jie Zhang, Xun Song, Chuen-Fai Ku, Juan Zou, Ji-Xin Li, Li-Jun Rong, Lu-Tai Pan and Hong-Jie Zhang

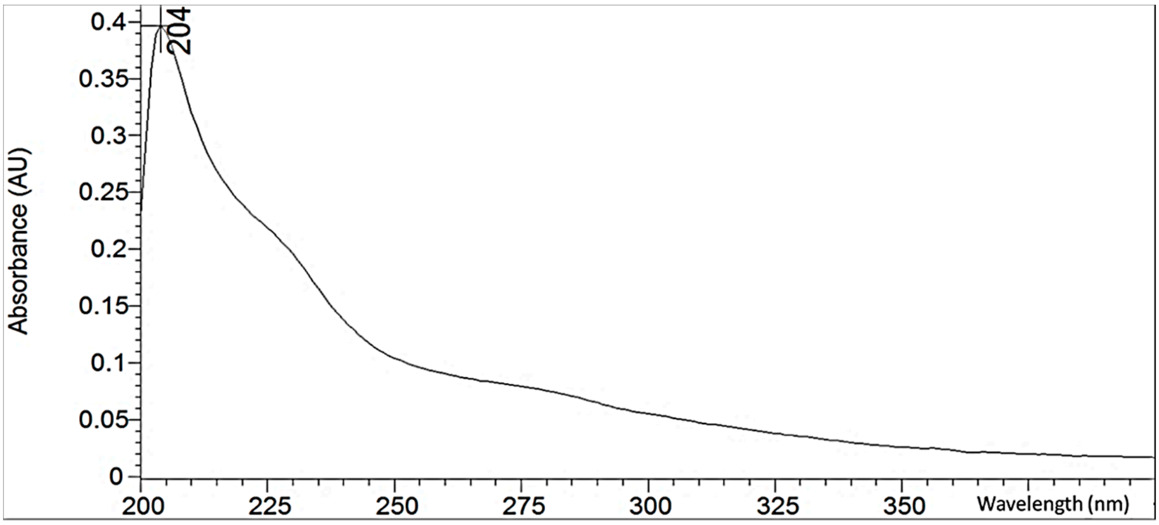

#	Name	Peaks (nm)	Absorbance (AU)
1	PH-4	204.0	0.39626

Figure S1. UV spectrum of henrin A.

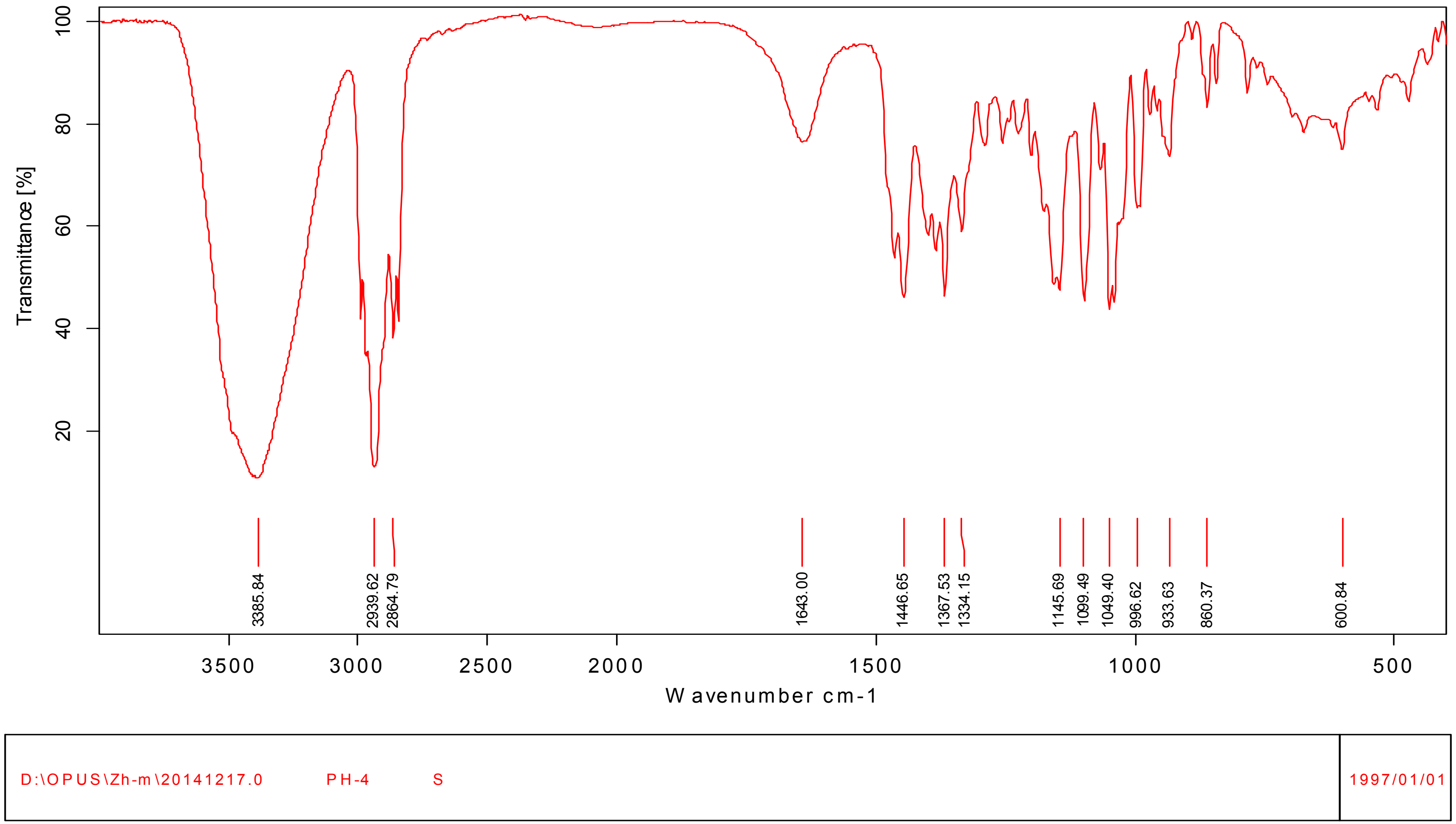

Figure S2. IR spectrum of henrin A.

Qualitative Analysis Report

**Data Filename**	fph4.d	**Sample Name**	fph4
**Sample Type**	Sample	**Position**	P1-A4
**Instrument Name**	Instrument 1	**User Name**	
**Acq Method**	SIBU.m	**Acquired Time**	12/23/2014 2:48:37 PM
**IRM Calibration Status**	Success	**DA Method**	Default.m
**Comment**			

**Sample Group**		**Info.**
**Acquisition SW Version**	6200 series TOF/6500 series Q-TOF B.05.01 (B5125.2)	

User Spectra

Fragmentor Voltage	Collision Energy	Ionization Mode
135	0	ESI

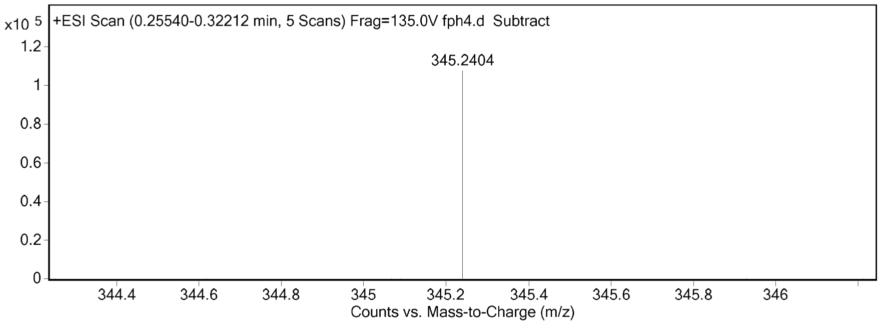

Peak List

m/z	z	Abund	Formula	Ion
301.1411	1	3629.4		
340.2841	1	2658.42		
345.2404	1	107610.23	C20 H34 O3	(M+Na)+
346.2436	1	23939.52	C20 H34 O3	(M+Na)+
347.2466	1	3126.18	C20 H34 O3	(M+Na)+
386.2666	1	5900.76		
667.4926	1	158151.25		
668.4957	1	67172.92		
669.4982	1	15186.46		
670.5001	1	2883.41		

Formula Calculator Element Limits

Element	Min	Max
C	3	60
H	0	120
O	0	30

Formula Calculator Results

Formula	CalculatedMass	CalculatedMz	Mz	Diff. (mDa)	Diff. (ppm)	DBE
C20 H34 O3	322.2508	345.2400	345.2404	-0.3	-1.0	4.0000

--- End Of Report ---

Figure S3. HR-ESIMS spectrum of henrin A.

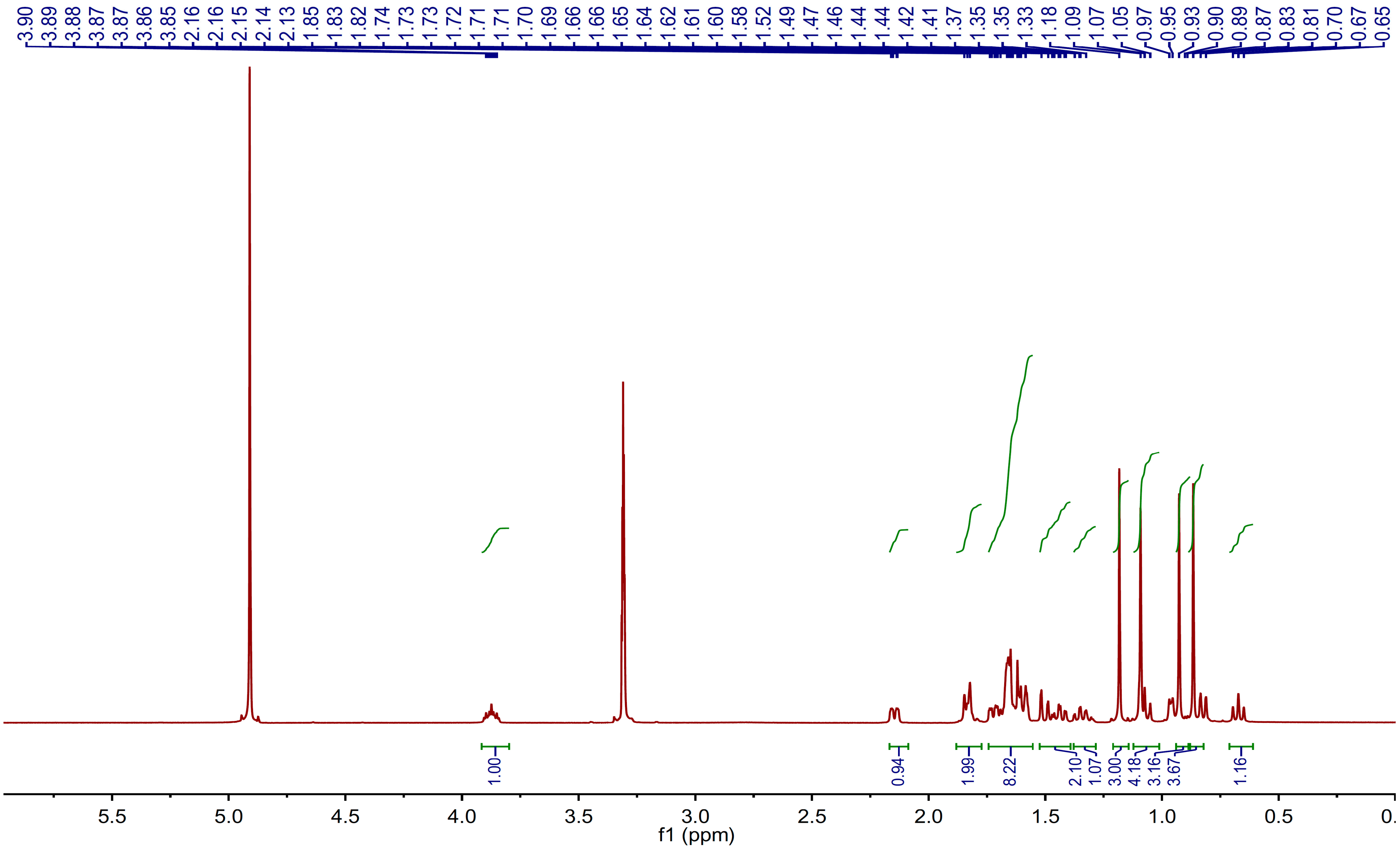

Figure S4. ^{1}H NMR (500 MHz, CD_3OD) spectrum of henrin A.

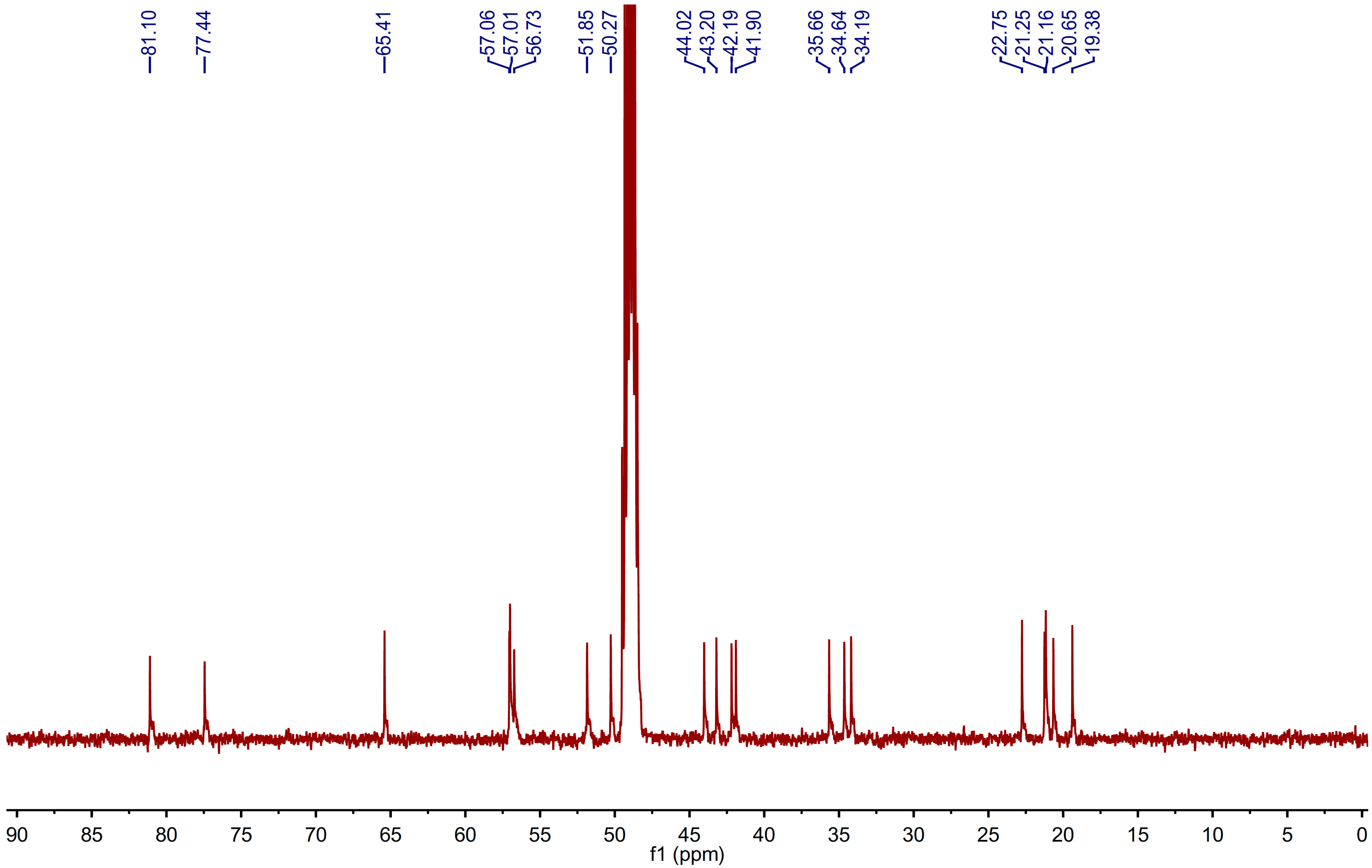

Figure S5. ^{13}C NMR (500 MHz, CD_3OD) spectrum of henrin A.

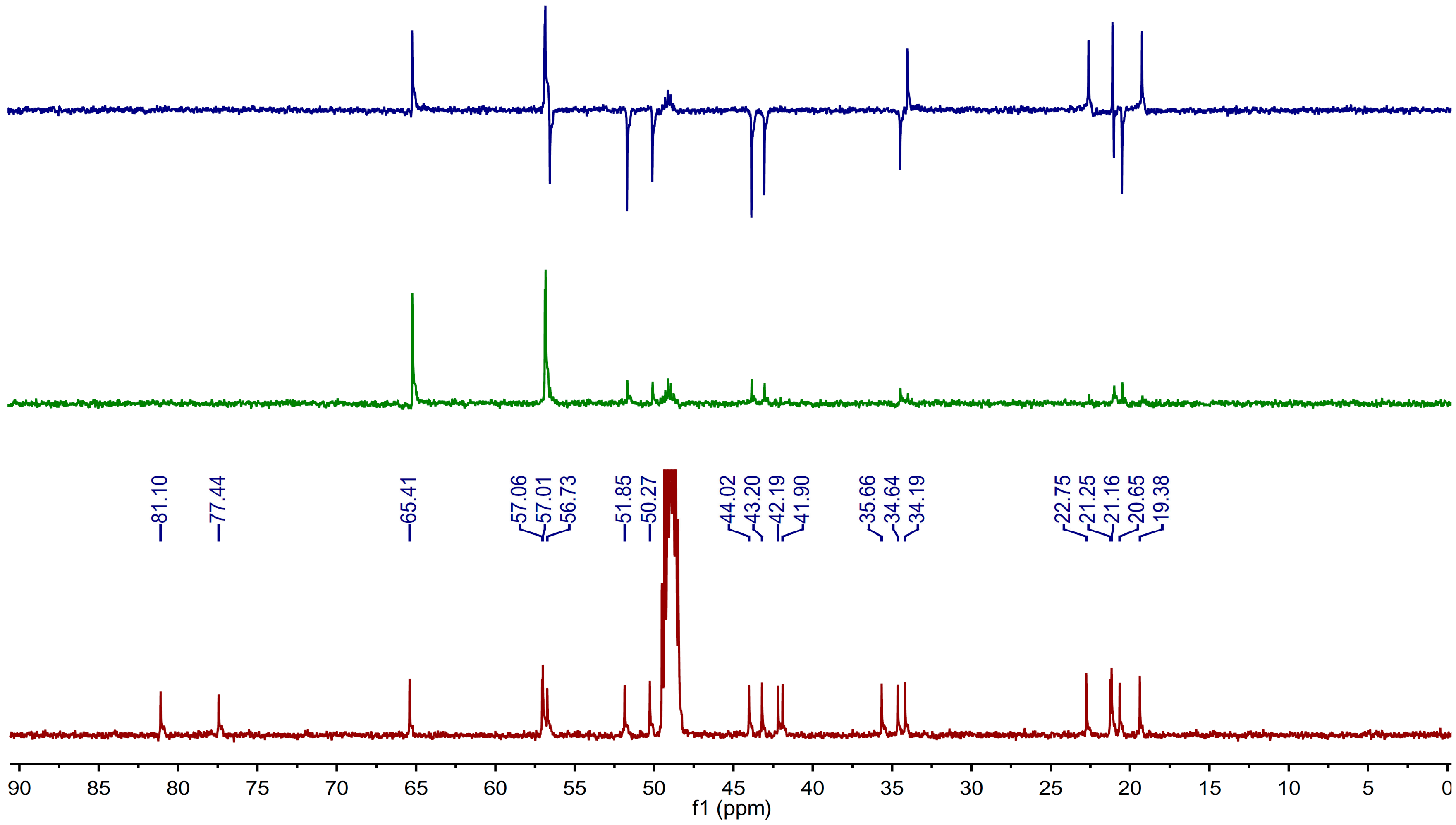

Figure S6. ^{13}C-DEPT (500 MHz, CD3OD) spectrum of henrin A.

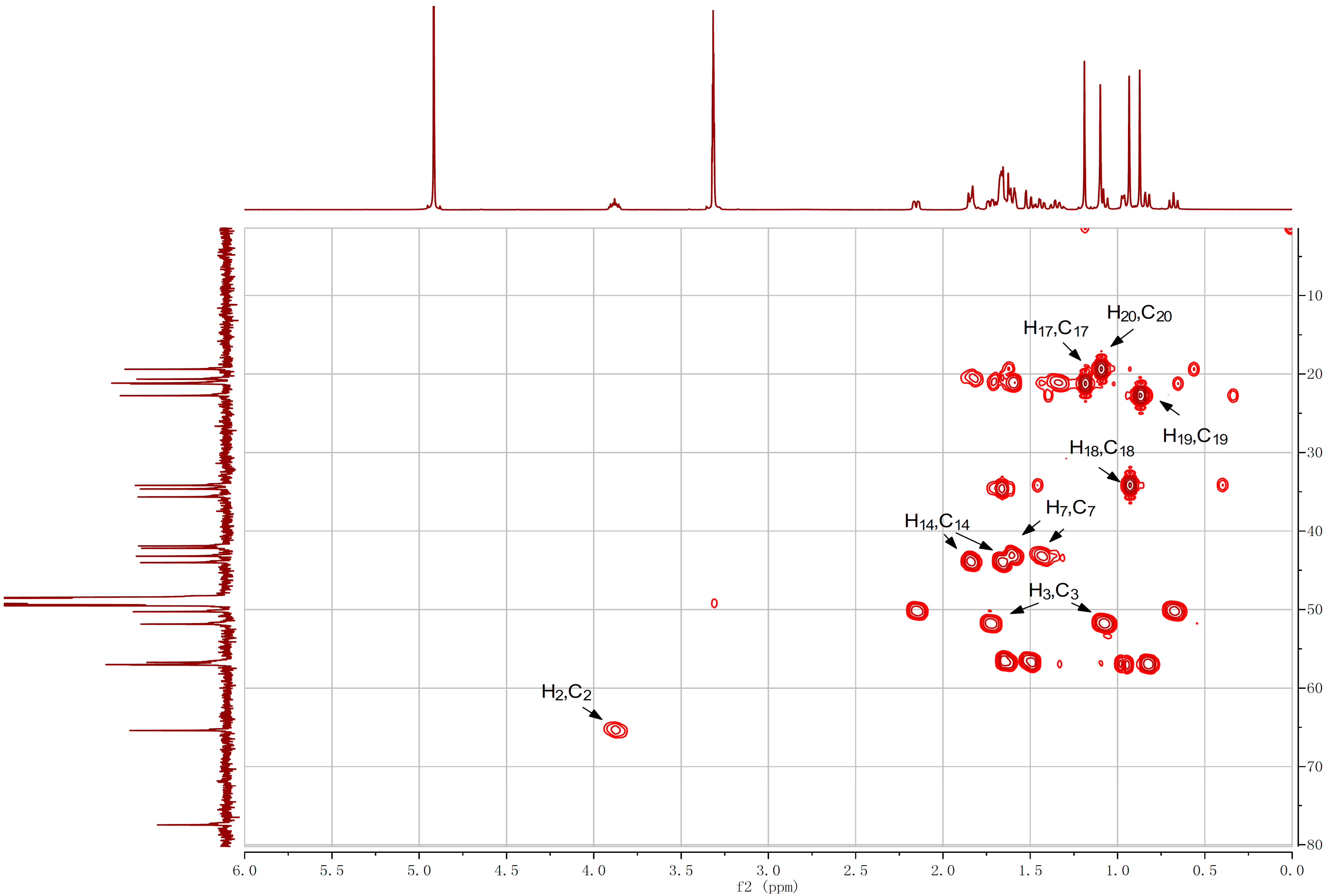

Figure S7. HMQC (CD3OD) spectrum of henrin A.

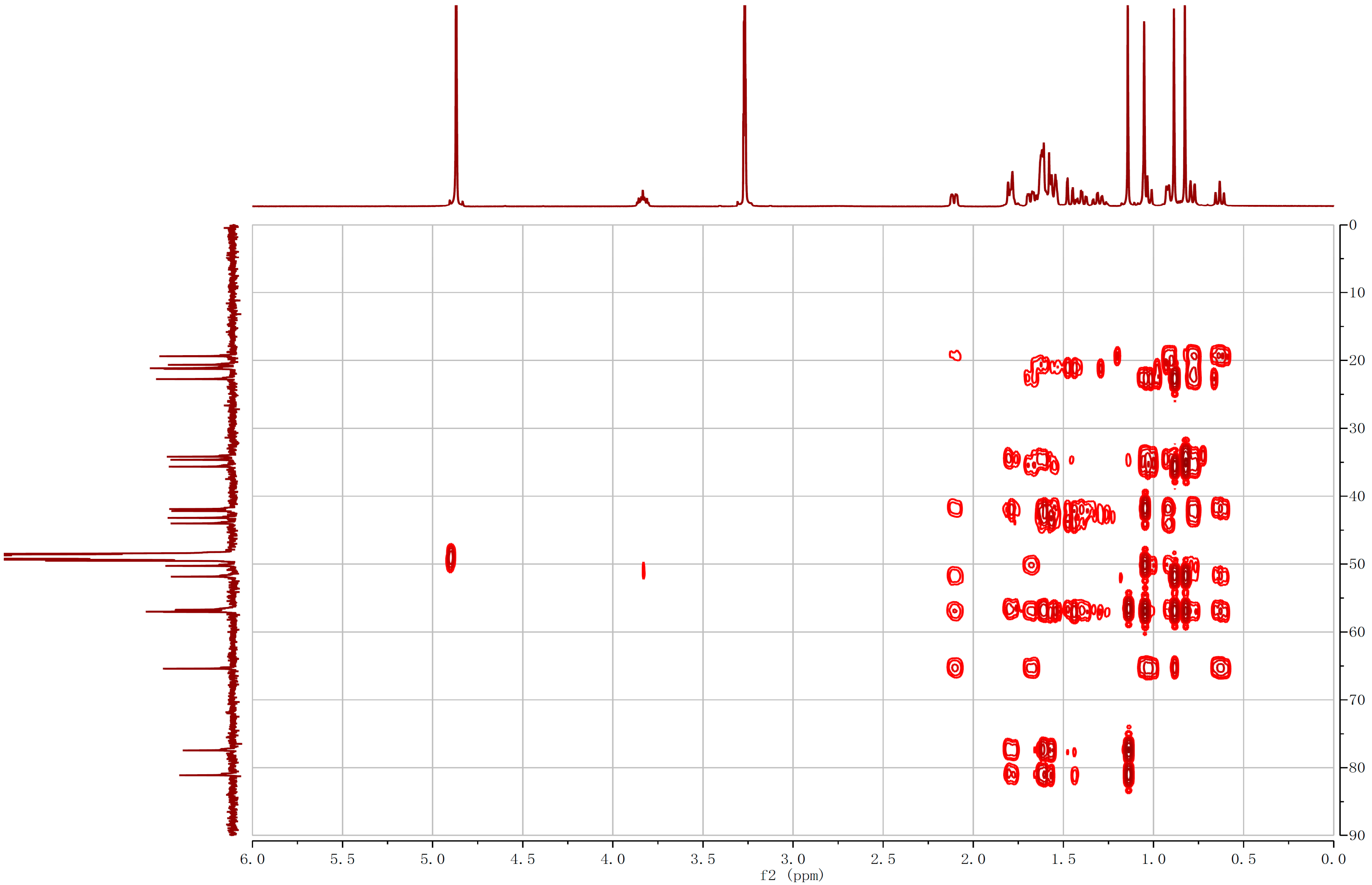

Figure S8. HMBC (CD_3OD) spectrum of henrin A.

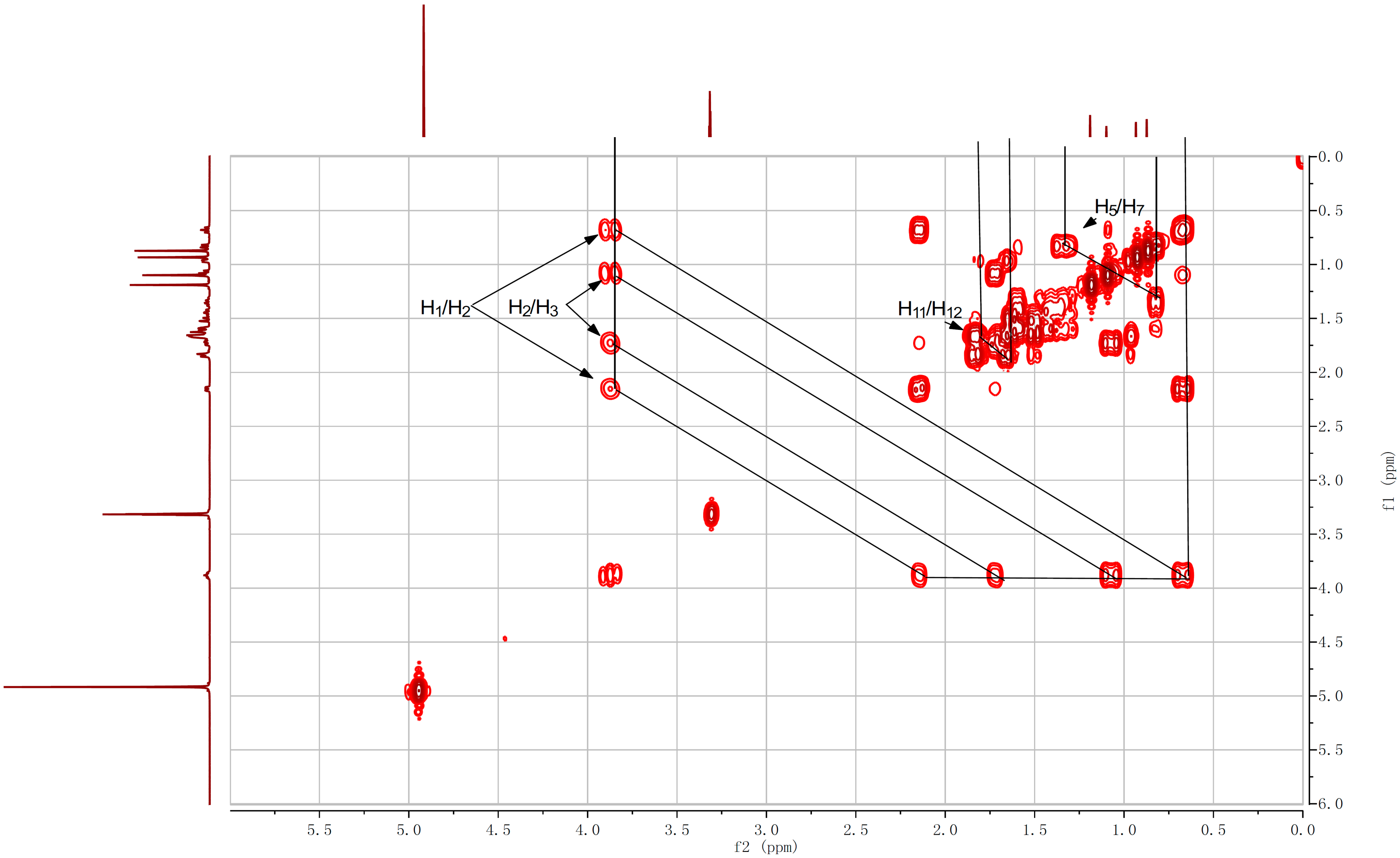

Figure S9. ^{1}H–^{1}H COSY (CD_3OD) spectrum of henrin A. The black lines show the correlations between the proton signals of the adjacent carbons.

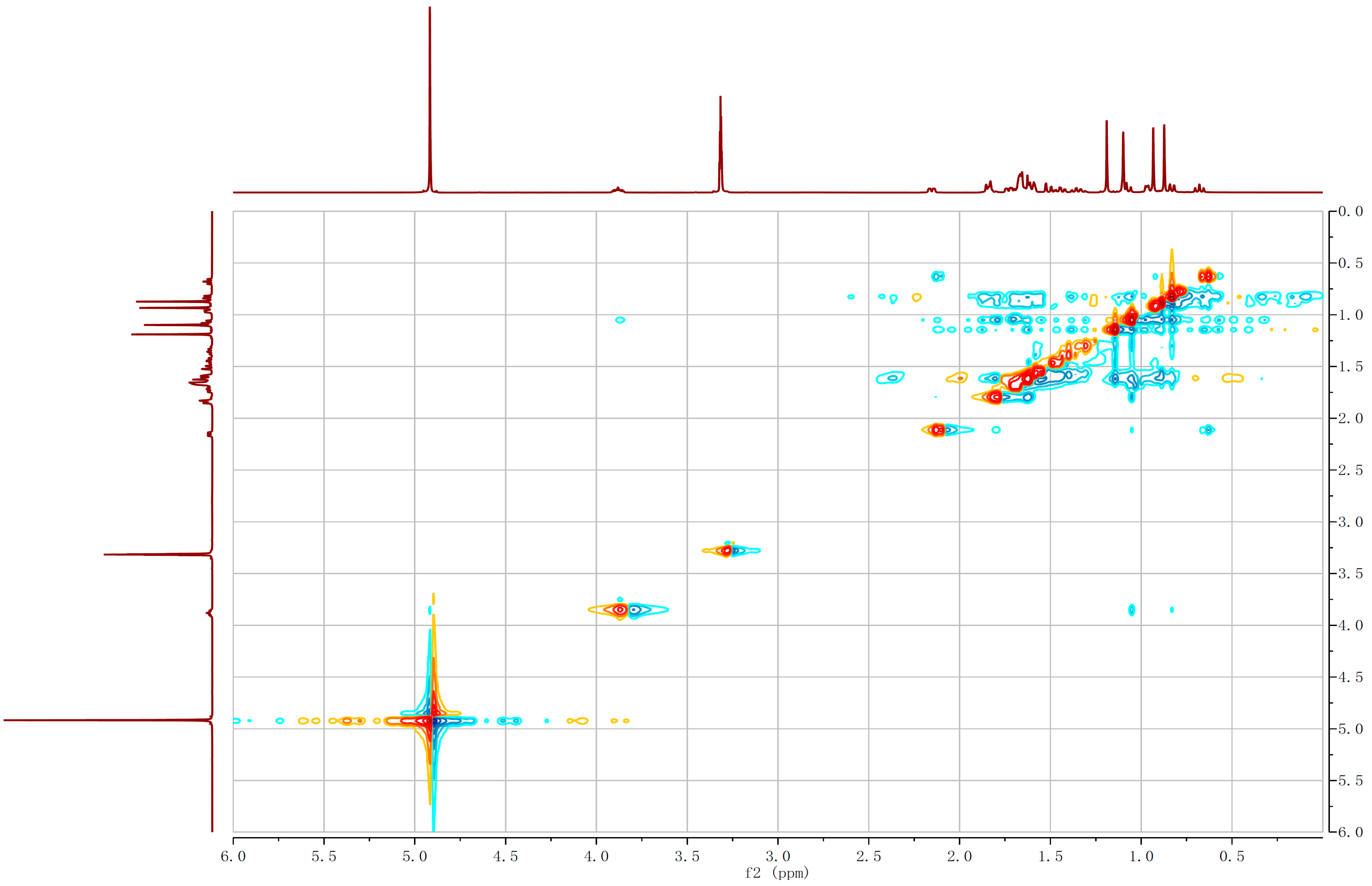

Figure S10. ROESY (CD_3OD) spectrum of henrin A.
